# Standardized *Polyalthia longifolia* leaf extract induces the apoptotic HeLa cells death via microRNA regulation: identification, validation, and therapeutic potential

**DOI:** 10.3389/fphar.2023.1198425

**Published:** 2023-08-24

**Authors:** Soundararajan Vijayarathna, Chern Ein Oon, Majid Al-Zahrani, Muyassar H. Abualreesh, Yeng Chen, Jagat R. Kanwar, Sumaira Sahreen, Shakira Ghazanfar, Mohd Adnan, Sreenivasan Sasidharan

**Affiliations:** ^1^ Institute for Research in Molecular Medicine (INFORMM), Universiti Sains Malaysia, Pulau Pinang, Malaysia; ^2^ Biological Sciences Department, College of Science and Arts, King Abdulaziz University, Rabigh, Saudi Arabia; ^3^ Department of Marine Biology, Faculty of Marine Sciences, King Abdulaziz University, Jeddah, Saudi Arabia; ^4^ Department of Oral and Craniofacial Sciences, Faculty of Dentistry, University of Malaya, Kuala Lumpur, Malaysia; ^5^ Department of Biochemistry, All India Institute of Medical Sciences (AIIMS), Bilaspur, India; ^6^ National Institute of Genomics and Advanced Biotechnology (NIGAB), National Agriculture Research Centre (NARC), Islamabad, Pakistan; ^7^ Department of Biology, College of Science, University of Ha’il, Ha’il, Saudi Arabia

**Keywords:** miRNA, apoptosis, gene ontology, anticancer, *Polyalthia longifolia*, standardized extract

## Abstract

*Polyalthia longifolia* var. angustifolia Thw. (Annonaceae), is a famous traditional medicinal plant in Asia. Ample data specifies that the medicinal plant *P. longifolia* has anticancer activity; however, the detailed mechanisms of action still need to be well studied. Recent studies have revealed the cytotoxicity potential of *P. longifolia* leaf against HeLa cells. Therefore, the current study was conducted to examine the regulation of miRNAs in HeLa cancer cells treated with the standardized *P. longifolia* methanolic leaf extract (PLME). The regulation of miRNAs in HeLa cancer cells treated with the standardized PLME extract was studied through Illumina, Hi-Seq. 2000 platform of Next-Generation Sequencing (NGS) and various *in silico* bioinformatics tools. The PLME treatment regulated a subset of miRNAs in HeLa cells. Interestingly, the PLME treatment against HeLa cancer cells identified 10 upregulated and 43 downregulated (*p* < 0.05) miRNAs associated with apoptosis induction. Gene ontology (GO) term analysis indicated that PLME induces cell death in HeLa cells by inducing the pro-apoptotic genes. Moreover, the downregulated oncomiRs modulated by PLME treatment in HeLa cells were identified, targeting apoptosis-related genes through gene ontology and pathway analysis. The LC–ESI–MS/MS analysis identified the presence of Vidarabine and Anandamide compounds that were previously reported to exhibit anticancer activity. The findings of this study obviously linked the cell cytotoxicity effect of PLME treatment against the HeLa cells with regulating various miRNAs expression related to apoptosis induction in the HeLa cells. PLME treatment induced apoptotic HeLa cell death mechanism by regulating multiple miRNAs. The identified miRNAs regulated by PLME may provide further insight into the mechanisms that play a critical role in cervical cancer, as well as novel ideas regarding gene therapeutic strategies.

## 1 Introduction

Cervical cancer remains a main cancer-related health problem despite much preventive enhancement including screening and immunization since the early 1970s. With an approximation of 570,000 cases every year, cervical cancer is the fourth most prevalent cause of death globally, with a significant number of occurrences among low-income populations and rural zones (Singh et al., 2011; Arbyn et al., 2020). Moreover, in 42 low-income nations, cervical cancer was the most common cancer in women (Arbyn et al., 2011). In 2008, around 530,000 women were diagnosed with invasive cervical cancer globally and 275,000 women passed away from cervical cancer (Cancer, 2007; McGraw and Ferrante, 2014). Several effective cervical cancer drugs have been expanding onto the market; cisplatin, cyclophosphamide, ifosfamide, doxorubicin, bleomycin, and neomycin, yet up until now, prominent drawbacks still cause incongruous results (Ye et al., 2014). When patients at early stages endure surgery, infertility befalls the younger ones while others simply undergo relapse due to the pertinacious, revertive, and metastatic abilities of cancer (Yaoxian et al., 2013).

MicroRNAs (miRNAs) are emerging classes of non-coding RNA that can significantly serve as therapeutic, diagnostic, and prognostic tools for cancer treatment (Lee et al., 2016; Ye et al., 2016) with customary key roles in the progression of cancer, notably in cervical cancer. These single-stranded miRNAs function by targeting mRNA through partial binding to their seed regions, thus hindering protein translation and obstructing gene expressions. Likewise, miRNAs had been also identified in the regulation of cell cycle, proliferation, migration and apoptosis of normal mammalian cells. Several miRNAs equivalently have also been reported to be dysregulated in cervical cancer tissues in relation to normal tissues (He et al., 2016).

Natural phytochemical compounds from numerous parts of medicinal plants such as leaf, flower, stem, and root are considered bioactive non-nutrient components. In many dietary kinds of research, the eating of green vegetables and fresh fruits was revealed to have an excellent defensive outcome against cancer and various diseases (Sun et al., 2002). The risk of cancer is identified to be two-fold greater in individuals with less consumption of vegetables and fruits than in those with increased consumption (Farvid et al., 2019; Wu et al., 2019). In this current research, we carefully studied the effects *P. longifolia* var. angustifolia Thw. (Annonaceae) standardized leaf extract on miRNA expression in HeLa cancer cells to induce apoptotic cell death. *P. longifolia* is an essential natural medicinal flora and is found throughout Sri Lanka, tropical parts of India, and Malaysia. Phytochemicals, namely, alkaloids, steroids, diterpenes, and various lactones, have been purified from *P. longifolia* bark. The extract and isolated compounds from *P. longifolia* stem bark were researched for multiple biological activities, such as cytotoxicity, antibacterial, and antifungal (Gautam et al., 2010; Jothy et al., 2016). Various biological activities of this medicinal plant were recently reported in literature owing to antioxidant, hepatoprotective (Jothy et al., 2012), genoprotective, acute oral toxicity (Jothy et al., 2013), and *in vivo* radioprotective activities (Jothy et al., 2016). Various bioactive compounds, including phenolic compounds, were isolated from this plant, namely, quercetin, quercetin-3-O-β-glucopyranoside, kaempferol-3-O-α-rhamnopyranosyl- (1→6)-β-galactopyranoside, kaempferol-3-O-α-rhamnopyranosyl- (1→6)-β-glucopyranoside, rutin, and allantoin (Sashidhara et al., 2011). Moreover, *P. longifolia* is also commonly applied in folk medicine as a tonic and febrifuge (Krishnamurthi, 1969). Our new comprehensive research disclosed that the standardized methanolic leaf extract of *P. longifolia* (PLME) has triggered the apoptotic HeLa cells death, mitochondrial membrane potential depolarization, and HeLa cell cycle arrest by controlling the redox status in HeLa cells (Vijayarathna et al., 2017a). Dietary anti-cancer agents such as resveratrol, camptothecin, and curcumin (Zhang et al., 2010; Zeng et al., 2012; Venkatadri et al., 2016) were demonstrated to modulate miRNAs in inducing cell death via apoptosis in many cancer cells. In that event, HeLa cells treated with PLME extract also similarly induced apoptosis as reported earlier (Vijayarathna et al., 2017a). Although the newest studies have shown that PLME can inhibit the growth of HeLa cells via apoptosis, the underlying cytotoxicity mechanisms and whether PLME treatment specifically regulates the miRNA in HeLa cells have never been studied in detail. Therefore, this study was conducted to reveal the regulation of miRNAs and their annotated functional roles in apoptosis and anti-proliferation effects in PLME-treated HeLa cells.

## 2 Materials and methods

### 2.1 Plant material and extraction

The fresh, mature leaves of *P. longifolia* were collected from Universiti Sains Malaysia and validated at the Herbarium of the School of Biological Sciences, Universiti Sains Malaysia, Pulau Pinang, Malaysia, where a sample of voucher specimen was deposited (Voucher specimen number: USM/HERBARIUM/11306). Before oven-drying at 30°C for 7 days, the leaf was cut into small sections and washed with purified water. Subsequently, the oven-dried leaf sample was grounded into fine powder by using an electronic grinder. A hundred grams of P. longifolia leaf powder was soaked in 400 mL of methanol at RT (23°C ± 2) for 7 days. After 7 days, the obtained filtrate was further concentrated in a vacuum rotary evaporator (Buchi, Switzerland) at 40°C. The concentrated filtrate was finally brought to complete dryness at 40°C in an oven in glass Petri dishes. The complete dry leaf extract paste was stored at RT in the dark. The measurement of rutin was accomplished by using the LC-MS/MS system for standardization purposes since this compound was regularly used as a chemical marker for standardization purposes in our laboratory. The rutin measure in PLME extract was established on the peak area calculated from the calibration curve equation of commercially available rutin compound (standard) (y = 275885x, r2 = 0.9977) as previously reported by Jothy et al. (Jothy et al., 2016). The amount of rutin in the PLME was found to be 8.83 µg (0.883%) in 1,000 µg.

### 2.2 Cell culture

Human cervical cancer cell HeLa from American Tissue Culture Collection (ATCC, United States) with cell passage number 15 was grown in Dulbecco Modified Eagle Medium (DMEM) supplemented with 10% Fetal Bovine Serum (FBS), glutamine (2 mM), penicillin (100 units/mL) and streptomycin (100 µg/mL). Viable cells were quantified using trypan blue dye and a hemocytometer. The number of cells was adjusted to 1.0 × 10^5^ cells/mL using DMEM supplemented medium and cultured at 37°C in a humidified 5% CO_2_ incubator.

### 2.3 Preparation of PLME treatment

The PLME IC_50_ concentration of 22.00 µg/mL used in this study to treat the HeLa cells has resulted from previous MTT and CyQUANT cytotoxicity assays (Vijayarathna et al., 2017b). The preparation of the PLME sample for treatment was accomplished by dissolving the extract in sterile-filtered Dimethyl sulfoxide (DMSO) (0.02% (v/v) in the culture medium) before addition to the culture media. The law concentration of solvent DMSO at 0.02% (v/v) was used in this study to avoid direct toxicity by DMSO against HeLa cells when used as a vehicle in this study. The HeLa cell was then treated with PLME extract at a 22.00 µg/mL of IC_50_ concentration. Similar cells cultured without the PLME treatment but treated in the same volume of 0.02% DMSO were prepared and regarded as an untreated group (negative control). Each experiment was carried out in triplicates.

### 2.4 Total RNA isolation and evaluation from HeLa cells

Total RNA was extracted from HeLa cells using Cytoplasmic and Nuclear RNA Purification Kit (Norgen Biotek, Thorold, Canada) according to manufacturer’s instructions and were analyzed for their concentration and quality based upon 260 nm/280 nm and 260 nm/230 nm absorbance ratios. A volume of 1 µL was used in the NanoDrop ND-1000 Spectrophotometer (Thermo Fisher Scientific) for each sample. RNA integrity was evaluated for 28S and 18S rRNA bands from 5 µL of total RNA on 1.0% agarose gel electrophoresis. The gel was stained with ethidium bromide and visualized with UV light. The images were captured using Vilber Lourmat (France). Then, Agilent 2100 Bioanalyzer (Agilent Technologies, Santa Clara, CA), was used to determine the integrity and the quality of RNA by indicating a strong ratio between ribosomal 28S and 18S RNA peaks fluorescence. Generally, A260:A280 and A260:A230 ratios are used to determine protein contamination in RNA samples. Pure RNA would indicate A260:A280 ratios between 1.8 and 2.1. A lower ratio will indicate protein contamination. Parallel to this, the A260:A230 ratio refers to the presence of organic contaminants where an ideal RNA purification should be closer to 2.0. A ratio above 1.97 is considered to be highly pure with minimized contaminations. Samples of total RNA with the integrity number RIN above 9 were further used for RNA sequencing. All samples were stored at −80°C in a deep freezer.

### 2.5 Construction and evaluation of small RNA (smRNA) library for deep sequencing

The preparation of the library was performed using Illumina^®^ TruSeq^®^ Small RNA Library Prep Kit (Illumina, Part # 15004197 Rev. G) for small non-coding RNAs. The libraries were constructed from the total RNA (5 μg) according to the manufacturer’s protocol. Briefly, Sequential ligation using blunt-ended adapters (3’ adapter and 5’ adapter) was ligated to total RNA using T4 RNA Ligase 2, Deletion Mutant (Epicentre), and T4 RNA Ligase (Epicentre) respectively. The enrichment of adapter-ligated RNAs was performed using RT-PCR. The cDNA was generated using SuperScript II Reverse Transcriptase (Life Technologies) and two primers that anneal to adapter-ends were based upon the generation of a single-end sequencing technique. The cDNA was then amplified with RP1 and RPIX (primers) using cycling conditions that consisted of an enzyme activation at 94°C for 30 s and then 11 cycles with denaturing at 98°C for 10 s, annealing at 60°C for 30 s and extension at 72°C for 15 s. Subsequently, the small RNA obtained from these libraries was then run on 6% Novex TBE gel electrophoretically for 60 min at 145 V, for gel electrophoresis purification. By using a sharp razor, the desired band sizes (135 bp to 160 bp) were cut out corresponding to the adapter-ligated constructs. The constructs within the gel were then purified from the gel and 3 µL from the total volume of each small RNA library was loaded into a DNA 1000 chip to be validated by using Agilent Technologies 2100 Bioanalyzer. The Illumina qPCR Quantification Protocol Guide was used to quantify the concentrations of libraries.

### 2.6 High-throughput sequencing

TruSeq PE Cluster Kit v3 (Illumina Inc., SA, United States) was used for cluster generation while TruSeq SBS Kit v3 (Illumina Inc., SA, United States) for sequenced using HiSeq 2000 sequencing system according to HiSeq 2000 System User Guide Part # 15011190 Rev.V HCS 2.2.38. Submission of FASTQ file per miRNA sequence was produced with HCS (HiSeq Control Software v2.2) for system control and base calling through integrated primary analysis software called RTA (Real Time Analysis. v1.18). Quality selected libraries were run in HiSeq 2000 using 100 bp PE (paired-end) reads. The BCL (base calls) binary is converted into FASTQ utilizing Illumina package bcl2fastq (v1.8.4) built with CASaVA software of Illumina. The files were subjected to the standard nomenclature of Illuimina as followed:

HELA_CONTROL_CCB_1.fastq.gz and HELA_TREATMNT_CCB_1.fastq.gz. The quality and integrity of Fastq files were verified using JAVA-based FASTqc (Babraham Institute of Bioinformatics, Cambridge).

### 2.7 MiRNA data processing and analysis

An important step to eliminate the low-quality bases and incorporate the adapter sequence was carried out with TrimGalore (http://www.bioinformatics.babraham.ac.uk/projects/trim_galore/) before the miRNA sequence alignment. Specific settings to trim Illumina small RNA adapter sequences of (TGGAATTCTCGG) with a stringency value of three were instructed and the resulting output files upon trimming completion were used to run fastQC. Further reads with a quality Phred score above 20 were retained. The quality reads were then aligned to sequences from built-in databases as below using Bowtie 1:

1. RepeatMasker—UCSC table browser (http://www.repeatmasker.org/)
2. rRNA (http://www.ensembl.org/biomart/martview/3b90656d3d4efe6e1f86b9fbd0 df1780)
3. tRNA—UCSC table browser (http://gtrnadb.ucsc.edu/)
4. miRBase version 21—(http://mirbase.org/)
5. RefSeq—UCSC table browser (https://www.ncbi.nlm.nih.gov/refseq/)
6. Genomic sequence (http://www.ncbi.nlm.nih.gov/projects/genome/assembly/grc/human/).

**FIGURE 1:**
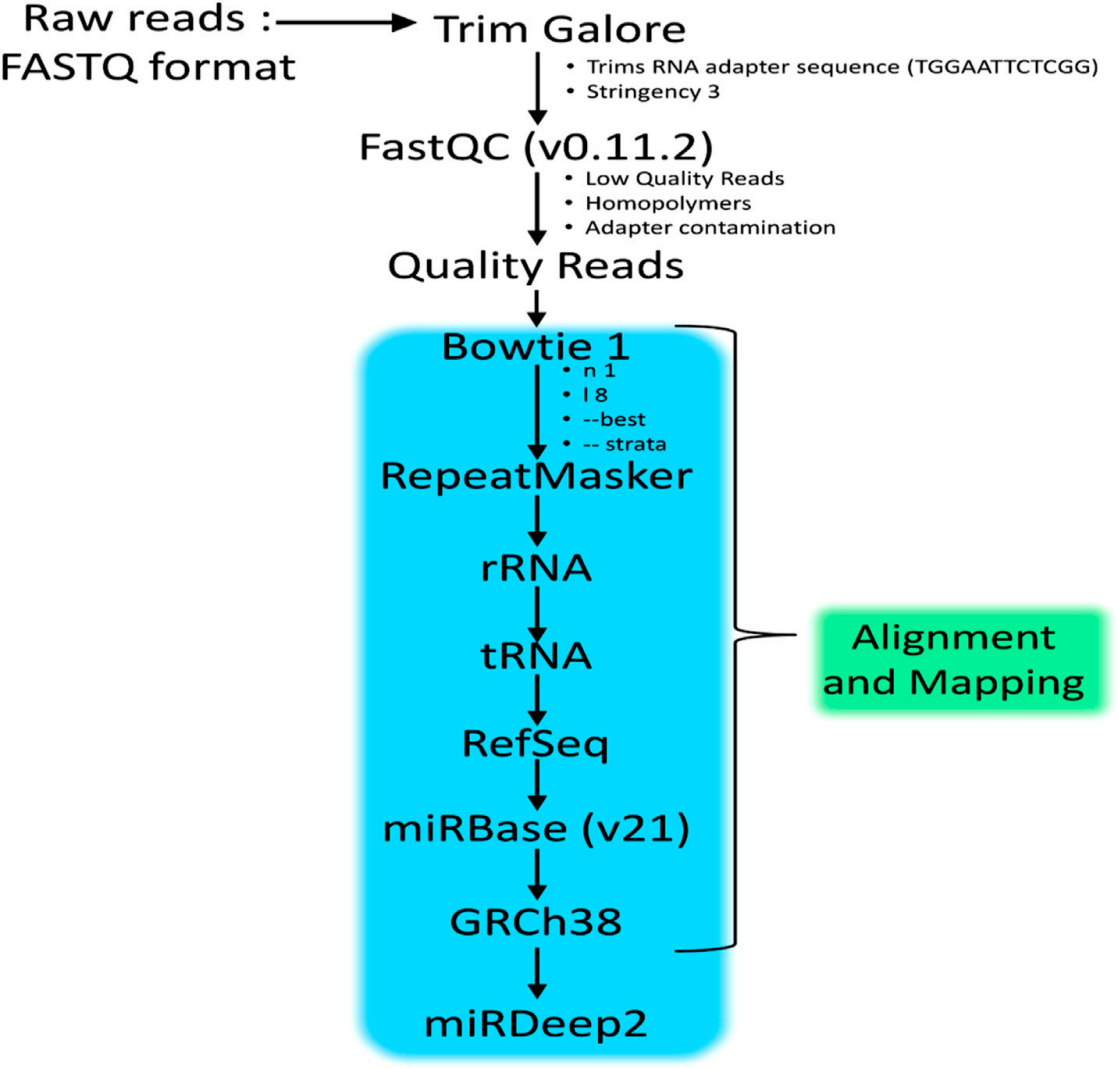
Workflow of the methodology adopted to identify the miRNAs in HeLa cells treated with *Polyalthia longifolia* methanolic extract (PLME) at IC_50_ concentration (22.00 µg/mL). (Protocol adapted from Genomax Technology Sdn. Bhd, Singapore).

**FIGURE 2:**
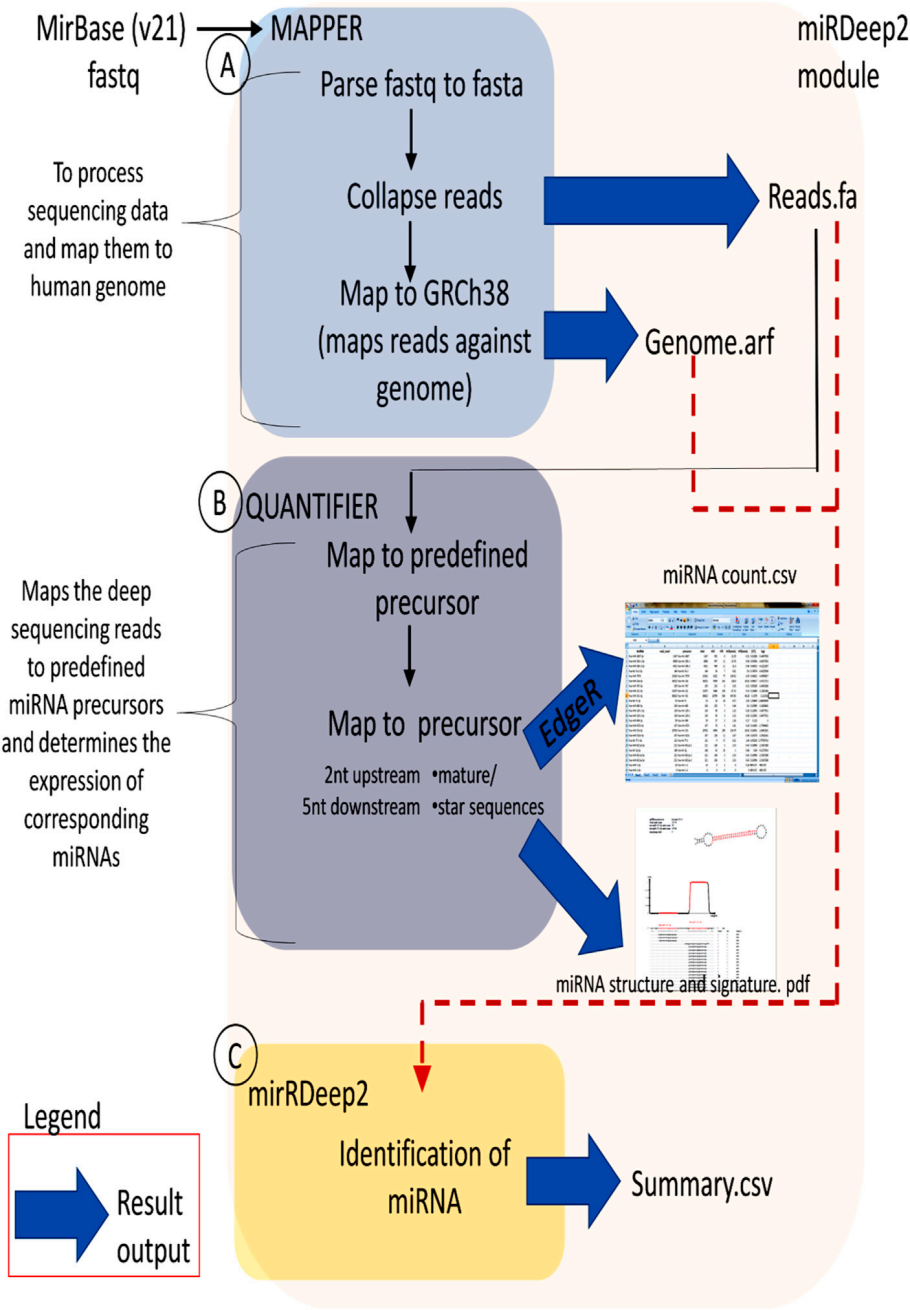
Flow chart of miRDeep2 module. **(A)** The Mapper module describes the mapping of Illumina output to the reference genome, **(B)** The Quantifier module quantifies read counts for miRNAs in sequencing data, and **(C)** The miRDeep2 module identifies miRNAs from the high-throughput sequencing data. For each module, the resulting output is shown (blue arrow). The file formats are fa, fasta; arf, arf mapping format, and csv, csv. Spreadsheet. (Protocol adapted from Genomax Technology Sdn. Bhd, Singapore).

Figure 1 illustrates the flow chart describing the methodology involved in the small RNA sequencing analysis. The Bowtie efficiency of mapping was based upon specific settings (-n1 - l8 –best-strata). The reads mapping to miRBase v21 is performed using mapped and unmapped reads from fastq along with SAM files from Bowtie mapper. The reads which are successfully mapped to miRBase v.21 are used in the quantification of expression analysis via miRDeep2 as described in Figure 2. MiRDeep2.pl is a wrapper function for the miRDeep2 program package. The script runs all necessary scripts of the miRDeep2 package to perform a deep sequencing data analysis for microRNA detection. The quantification was done via mapper.pl and then quantifier.pl from the miRDeep2 software package with the settings of (-h -m); explained in detailed–h (parse fastq to fasta format which is needed for miRDeep2.pl) and–m (Collapse reads which is needed for miRDeep2.pl). The mapper.pl function produced collapsed fasta files and mapped the position of the reads to the GRCh38 genome as an “arf” file format, both of which were required in subsequent analysis in miRDeep2. After the collapsed fasta file and arf files were produced, the expression data for miRNA can be generated by the quantifier.pl software script from miRDeep2 with similar settings as Bowtie.

### 2.8 Analysis of miRNA expression profile

Before assessing differential expression, the count data for untreated and PLME-treated conditions were first normalized across their libraries. The samples were first normalized by calculating transcripts per millions of total RNA reads (TPM) and were utilized for comparing the relative abundance of miRNAs between each data set. The TPM was quantified with the formula below (He et al., 2015);

$$TPM=\left[\left(\frac{Number\;of\;Actual\;Reads}{Total\;Number\;of\;Clean\;Reads}\right)\right]\times 10^{6}$$

The miRNAs in PLME-treated HeLa cells were further correlated with the untreated to identify the differently expressed miRNAs.

In order to determine statistically significant differently expressed miRNAs, the R (version 2.14.1) Bioconductor package EdgeR (V2.4.6.) (Robinson et al., 2010) was applied. The EdgeR was utilized as it implements a negative binomial distribution model to separate biological from technical variation (Robinson et al., 2010) accounting for more count efficiency. Differently expressed miRNAs were interpreted as having a Benjamini and Hochberg (Benjamini and Hochberg, 1995) corrected *p*-value of <0.05.

MicroRNAs which are differentially expressed between the groups of untreated and PLME*-*treated HeLa cells were identified by calculating with an absolute log_2_ fold change as follows:

$${\mathrm{Log}}_{2}\left[\frac{Normalised\;Expression\;of\;PLME\;Teated\;HeLa\;Cells}{Normalised\;Expression\;of\;Untreated\;HeLa\;Cells}\right]$$

The criteria for significantly upregulated miRNAs were decided as fold change (log_2_) > 1 whereas fold change (log_2_) < −1 is chosen for downregulated miRNAs (Wang et al., 2014). Then the selected miRNAs were clustered according to their expression abundance in their 2 conditions.

### 2.9 Meta-analysis of miRNA expression data

#### 2.9.1 MiRNA-gene interaction analysis

The results obtained through the differential miRNA analysis (untreated and PLME treated HeLa cells), miRNAs, and their gene interaction were predicted miRGate (http://mirgate.bioinfo.cnio.es/miRGate/).

In miRGate, miRNA target predictions were computed using in-house prediction methods; miRanda, Pita, Rnahybrid, TargetScan, and Microtar, and then screened for their experimentally validated genes in order to understand the miRNA-UTR targets utilizing four different built–in databases of MirGate, namely,; TarBase 6.0, MirTarbase 4.5, miRecords and oncomiRDB. Default parameter settings were selected for each method. The target genes that represent the intersection of at least three algorithms with at least one experimentally validated database were selected as candidate target genes for further analysis.

#### 2.9.2 Gene ontology (GO) and pathway analysis

Gene ontology and pathway enrichment analysis were performed on significantly regulated miRNAs in PLME-treated HeLa cells compared to untreated HeLa cells to understand their biological role. For example, given a set of miRNAs that are upregulated, an enrichment analysis will find which GO terms are over-represented (or under-represented) using annotations for that miRNA. The Database for Annotation, Visualization, and Integrated Discovery (DAVID) Bioinformatics Resources 6.8 Beta (http://david.abcc.ncifcrf.gov/) was used to perform the Gene ontology and pathway analysis. On the DAVID open database homepage, the gene list manager panel was used to submit the gene list corresponding to the selected downregulated miRNAs obtained from miRGate analysis. The Identifiers were fixed as “OFFICIAL_GENE_ SYMBOL”, and the species used for annotation was selected as *Homo sapiens* before clicking the submit button. In order to control the behavior of fuzzy clustering in DAVID, the stringency was set at high. The enrichment annotation terms were determined by their enrichment score, *p*-value (or EASE score), fold change, Benjamini and Hochberg multiple test correction, and false discovery rate (FDR). A smaller *p*-value is considered enriched, and a *p*-value <0.05 were deemed significant, while FDR <0.01 is implied statistically significant. Furthermore, Enriched annotations and pathways were selected/ranked based on a combined score which was calculated by the EnrichR platform (http://amp.pharm.mssm.edu/Enrichr/) following Z-score permutation background correction on the Fischer Exact Test *p*-value.

### 2.10 Determination of bioactive compounds in PLME using LC-ESI-MS/MS

The Agilent 1,200 series Ultra-High-Performance Liquid Chromatography (UHPLC) system was used in conjunction with an Agilent 6520 Accurate-Mass quadrupole time of flight mass spectrometer (QTOF-MS) to identify the bioactive molecule (Agilent Technologies, United States). A vacuum solvent degassing device, a capillary pump, and an automated sample injector comprised the UHPLC system. With an m/z range of 100–3,200, the MS had an electrospray ionization (ESI) interface and could operate in both positive and negative modes. Fragmentor voltage 125 V; nebulizer pressure 45 psi; capillary voltage 3500 V; gas temperature 300°C, gas flow 10 L/min, and skimmer 65 V were the ESI conditions. Agilent Zorbax Eclipse XDB-C18, Narrow-Bore 2.1 × 150 mm, 3.5 microns (Agilent Technologies, United States) was used to perform the chromatography. The mobile phase was 0.1% formic acid in water (A) and 0.1% formic acid in acetonitrile (B), and the auto-sampler compartment was kept at 4°C. The multi-step linear gradient was applied as follows: 0 min, 5% B; 5 min, 5% B; 20 min, 100% B; 25 min, 100% B. Before the following analysis, the initial condition was kept for 5 min with 1 μL injection volume and 0.5 mL/min as the chosen flow rate.

## 3 Results

### 3.1. RNA integrity

**FIGURE 3:**
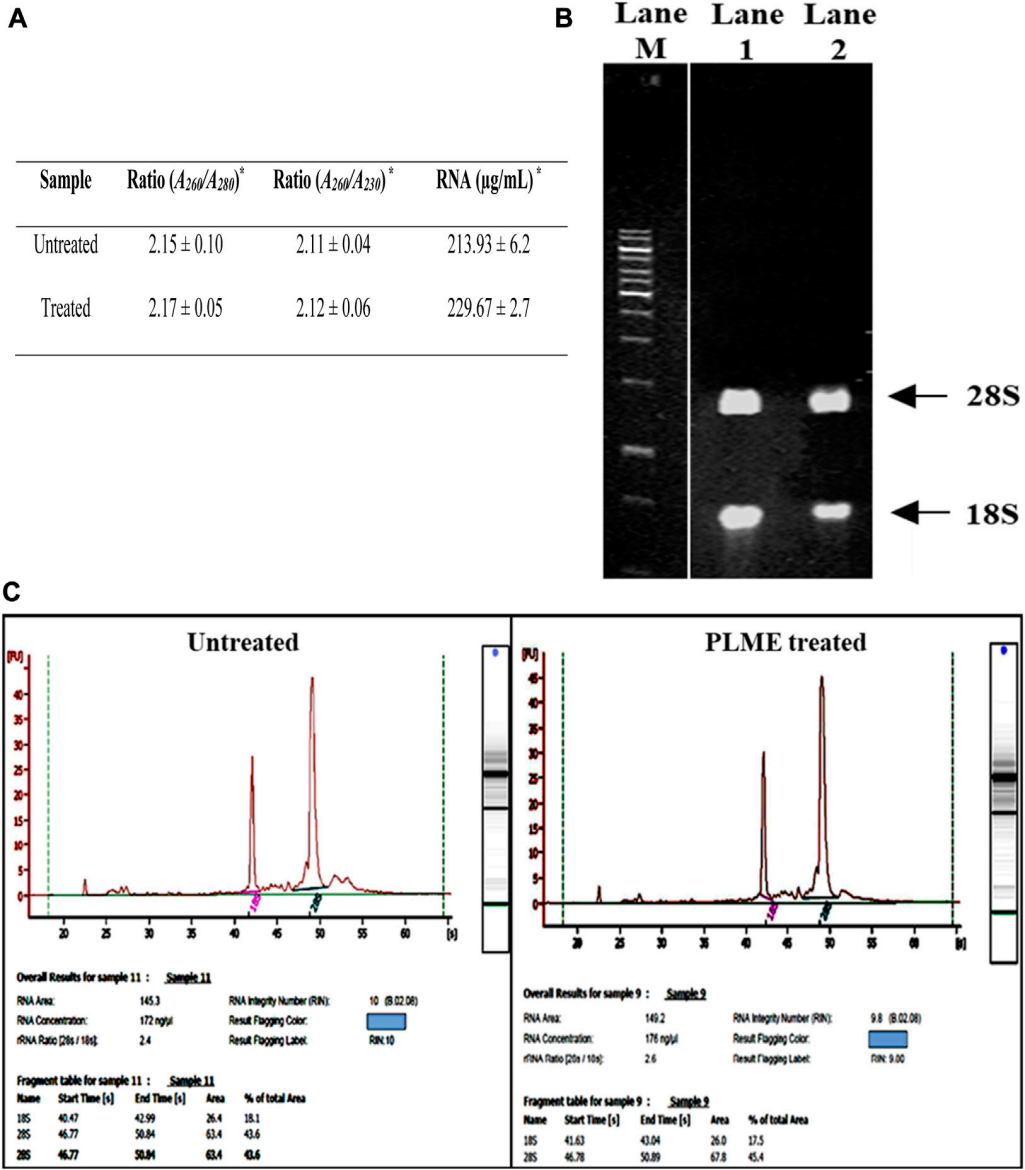
Determination of RNA integrity number of untreated and PLME-treated HeLa cells. **(A)** Spectrophotometer results for RNA concentration and purity control based on A260/A280 and A230/A280 ratios for untreated and PLME-treated HeLa cells. * Average value of triplicates. **(B)** Agarose gel containing RNA isolated from HeLa cells. Lane M; 1kb DNA marker, Lane 1; RNA purified from untreated cells and Lane 2; RNA purified from PLME treated cells. **(C)** RNA electrophoretic traces for untreated and PLME-treated cells. The images are representative of three independent experiments.

In this study, the spectrophotometer-based RNA concentration garnered from the untreated sample of HeLa was 213.93 ± 6.2 µg/mL with (*A*_*260:*_ *A*_*280*_) ratio of 2.15 ± 0.10 and (*A*_*260*_ *: A*_*230*_) ratio of 2.11 ± 0.04 (Figure 3A). Similarly, PLME treated HeLa cells, generated RNA concentration of 229.67 ± 2.7 µg/mL with readings of 2.17 ± 0.05 and 2.12 ± 0.06 respectively for (*A*_*260:*_ *A*_*280*_) and (*A*_*260:*_ *A*_*230*_) ratio. Figure 3B displays bands appearing on the gel, the typical pattern of those from 18S and 28S ribosomal (rRNA) species were found in extracted cytoplasmic RNA and there were also other faint bands observed underneath 18S bands where smaller RNA species were speculated. The integrity of RNA is considered high quality when the ratio of 28S:18S bands is approximately 2:1 or higher. Figure 3C indicates purified cytoplasmic RNA from untreated and PLME*-*treated HeLa cells which were evaluated using the Agilent software that accounts for the entire electrophoretic traces of RNA. The electrogram depicts the peaks from 28S and 18S where the presence of smaller peaks at the beginning of the electrogram denoted smaller RNA species. Sharp and taller peaks for 28S and 18S rRNA showed high integrity of RNA. The untreated sample achieved a RIN number of 10, with an RNA area of 145.3 and an rRNA (28S:18S) ratio of 2.4. The concentration obtained is 172 ng/µL. The PLME*-*treated HeLa cells also responded with a RIN number of 9.8, RNA area of 149.2, and rRNA (28S:18S) ratio of 2.6. The concentration measured is 176 ng/µL. The electropherograms provided by both samples are of high-quality RNA samples since there were visibly sharp, and possessed well-defined 28S and 18S peaks, with (28S:18S) ratio above 2. Apart from that, lower noise between these peaks and minimal low molecular weight contamination was also detected. Generally, these criteria are accepted for good-quality RNA.

### 3.2 Library quality assessment

**FIGURE 4:**
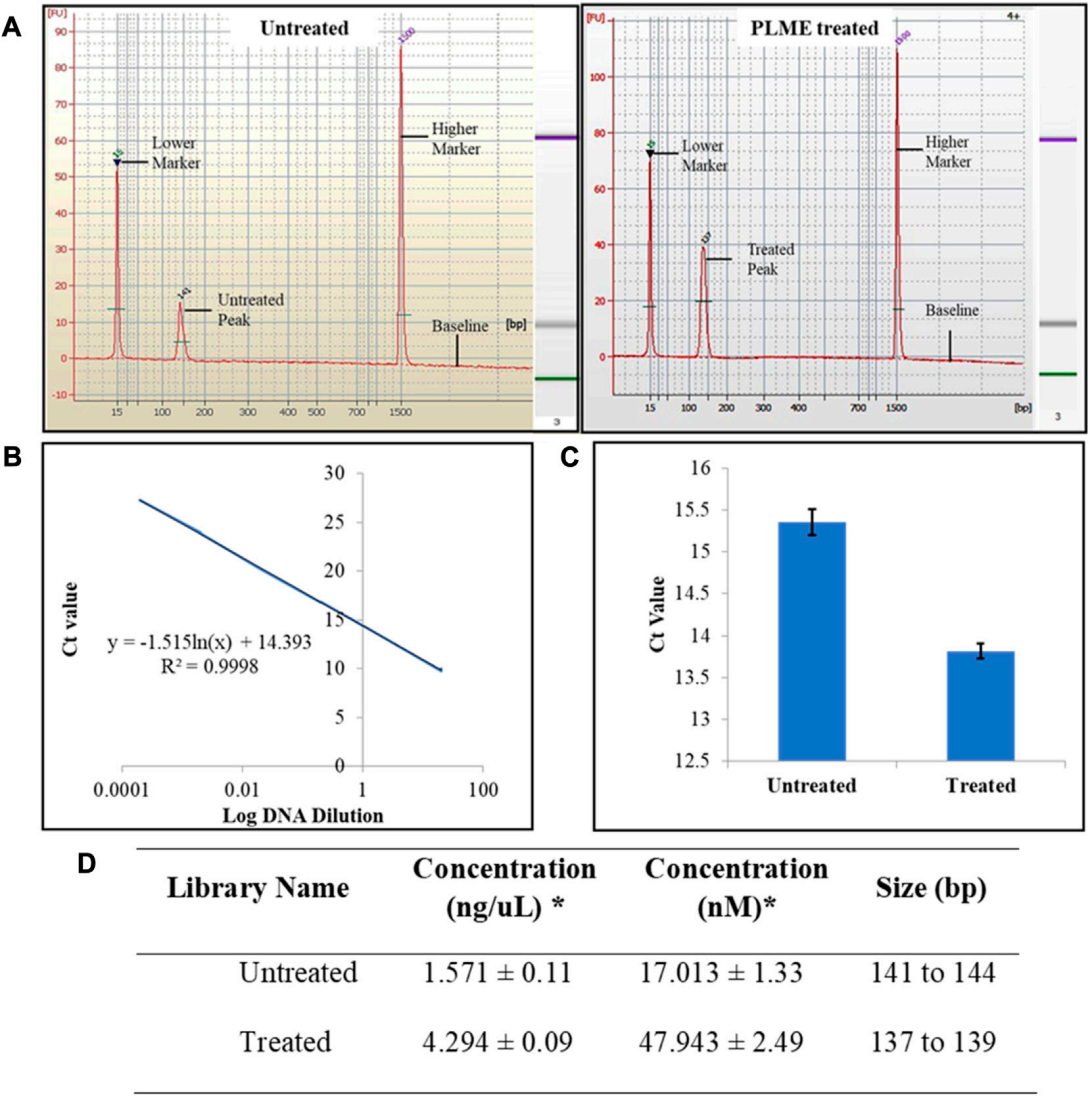
Small RNA library quality assessment. **(A)** Typical results from Agilent 2100 Bioanalyzer electropherogram trace of untreated and PLME treated exhibiting evidence of microRNA purified from their respective cDNA libraries. Images are representative of three independent experiments. **(B)** Standard graph Ct *versus* log DNA dilution. **(C)** Observed C_t_ values of untreated and PLME*-*treated HeLa cell libraries obtained from qPCR analysis. The graph represents average ±SD values of triplicate experiments. **(D)** Summary table of small RNA libraries quantity and base pair size. * Average value of triplicates.

Upon small RNA library amplification, the purified cDNAs were subjected to quality assessment. The purified size of PCR enriched fragments is checked with Agilent 2100 Bioanalyzer using Bioanalyzer DNA 1000 chip and the output was integrated into the form of electrophoregram. The electropherogram traces displayed in Figure 4A justified the presence of miRNA by corresponding to the peak at 141 at the concentration of 18.25 nM (untreated) and 137 bp at 48.81 nM (PLME treated) respectively. The markers are identified at the peaks of 15 bp and 1,500 bp. A gel-like image was also embedded next to the electropherogram for visualizing fragment sizing and distribution in Figure 4A. Referring to Figure 4B a standard graph was plotted between C_t_ values and log DNA dilution. The graph was quantified based on logarithmic trend and the resulting equation is defined as y = −1.515 ln(x) + 14.393, with an R^2^ value of 0.9998.

The C_t_ values for the respective libraries were determined by qPCR. Here in Figure 4C, the average C_t_ values were calculated as 15.35 ± 0. 15 (untreated) and 13.82 ± 0.09 (PLME). The table in Figure 4D contains the quantified concentration of the libraries along with their size base pairs. The untreated HeLa cells achieved a concentration of 1.571 ± 0.11 ng/uL with sizes of base pairs ranging from 141 to 144 whereas PLME-treated HeLa cells obtained a concentration of 4.294 ± 0.09 ng/uL with base pairs ranging from 137 to 139.

### 3.3 Pre-processing quality of raw reads

**FIGURE 5:**
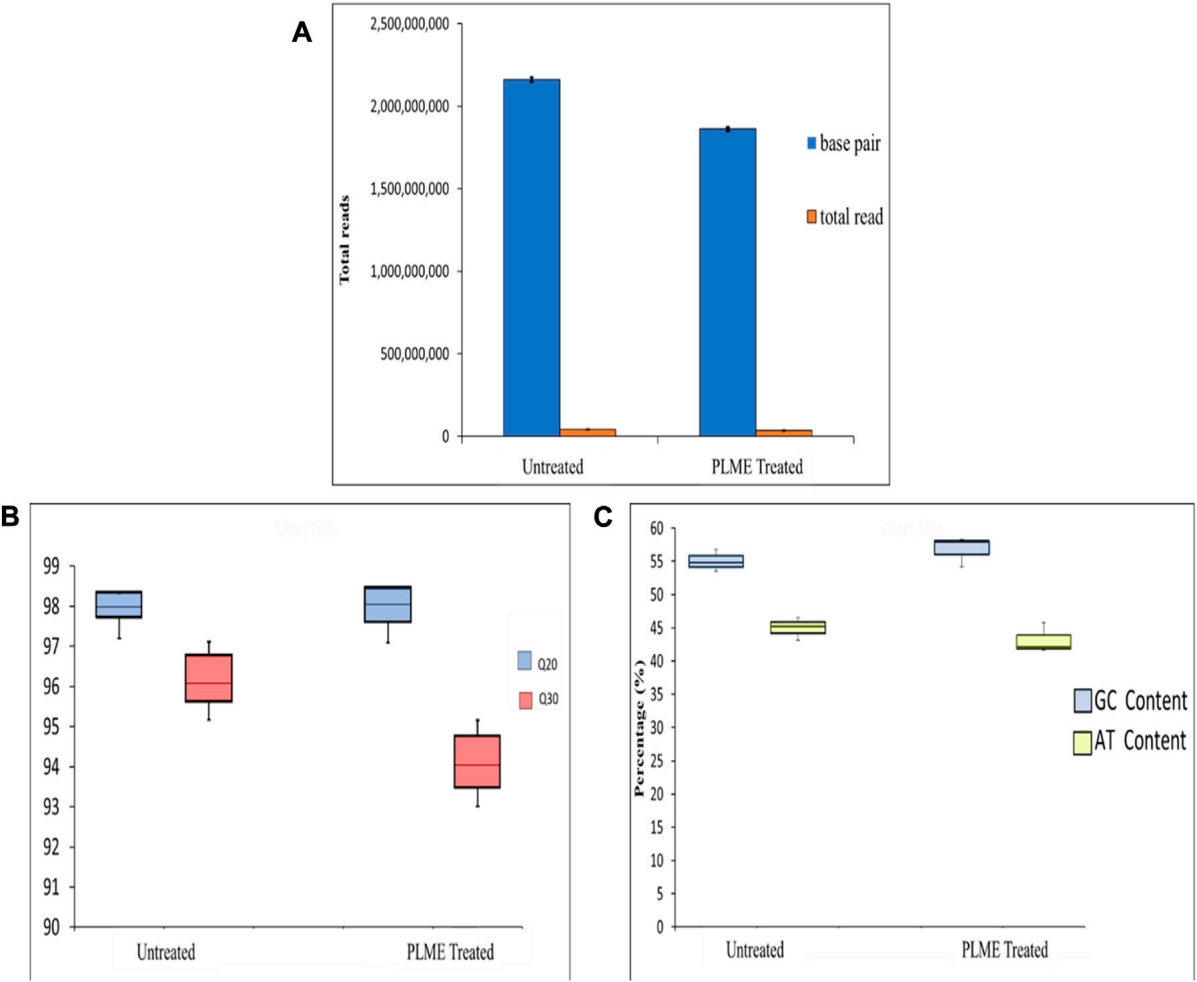
Quality control of the raw reads. **(A)** The total read of base pair and reads generated from untreated and PLME-treated HeLa cells. The graph represents the average ± SD of triplicate values. **(B)** Box-and-whiskers plot displaying GC and AT content from untreated and PLME-treated HeLa cells. **(C)** Box-and-whiskers plot exhibiting the Phred Q20 and Q30 quality produced by untreated and PLME-treated HeLa cells. The plots include median, upper quartile, lower quartile, maximum, and minimum triplicate values.

It is a crucial step to check for raw sequencing efficiency before the data is used for further interpretation and analysis. A few parameters that are assigned for this procedure include base quality, GC content, and Phred score (Figure 5). The total number of bases, total reads, GC (%), Q20 (%), and Q30 (%) were calculated for the two sets of samples. The untreated HeLa cells produced an average of 2,265,871,816 total read bases (bp) and 44,428,859 total reads. The GC content was measured to be 55.03% ± 0.97% with respective scores for Q20 and Q30 of 98.02% ± 0.36% and 96.25% ± 0.64%. The PLME*-*treated HeLa cells generated an average reading of 1,877,230,897 (bp) total bases with total reads of 36,808,449. The GC contents were measured to be 56.81% ± 1.32% with a Q20 score of 98.00% ± 0.48% and a Q30 of 94.16% ± 0.72%. The GC content (%) is exhibited in Figure 5 as box-and-whisker plots where the average GC content of these samples falls between the ranges of 55%–57% which is a remarkable sign that the reads are of good quality. A GC content of the human genome (100 kb) will have content as lower as 35%–60% (Lander et al., 2001). The Phred score had been a good indicator of raw read quality upon where the Phred scale demonstrated the probability *p* that the base call is incorrect. Figure 5C displays a distribution of Q20 and Q30 among both samples. The average scores above 90% prove the accuracy of the sequence data.

### 3.4 Transcriptome mapping analysis

**FIGURE 6:**
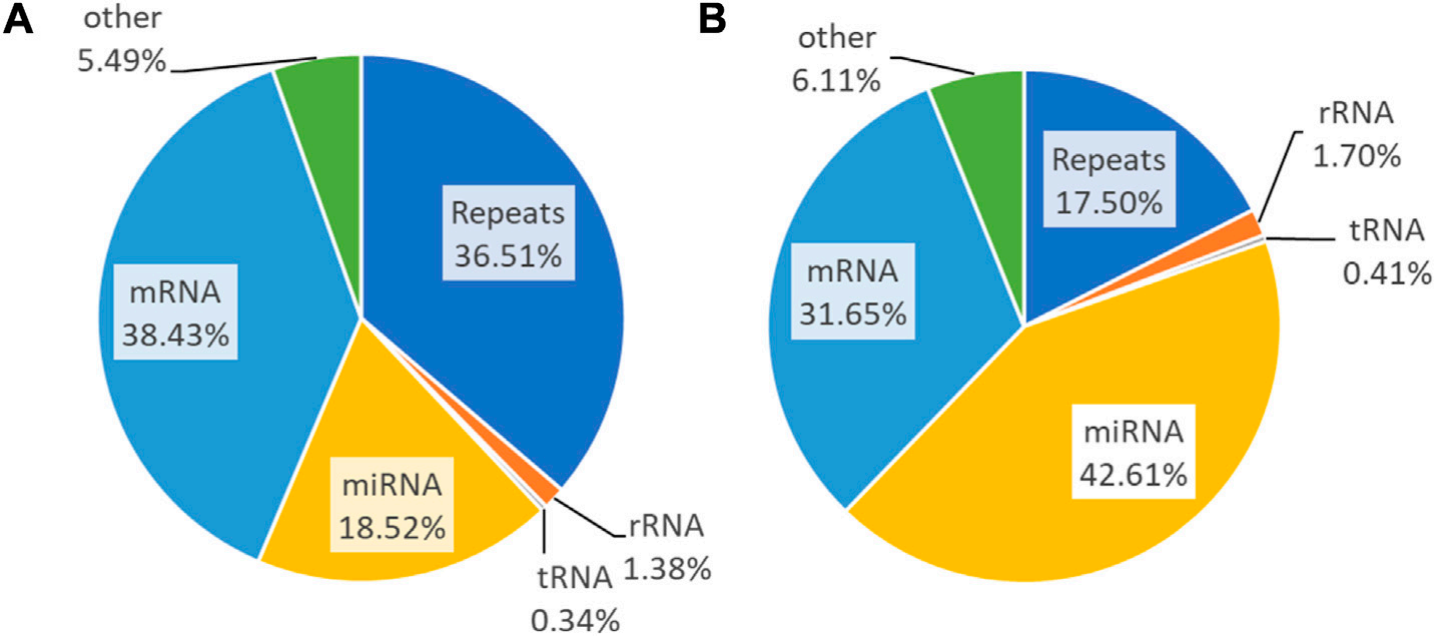
Small RNA sequencing read statistics of the **(A)** untreated and **(B)** PLME-treated HeLa cells. Pie charts represent the genomic distribution of the reads that were mapped to repeats, rRNA, tRNA, mRNA, and miRNA. Those unmapped are categorized as others.

Transcriptome reads of small RNAs are aligned to RepeatMasker to inspect redundancy and then sequentially mapped to rRNA, tRNA, miRNA, mRNA, and others. The untreated samples constitute repeats of 36.51%, 1.38% (rRNA), 0.34% (tRNA), 18.52% (miRNA), 38.43% (mRNA), and 5.49% (others) (Figure 6A). The PLME treated HeLa cells acquired percentages of 17.50%, 1.70%, 0.41%, 42.61%, 31.65%, and 6.11% respectively for repeats, rRNA, tRNA, miRNA, mRNA, and others (Figure 6B). It can be perceived that PLME treated cells possess higher percentage of miRNAs compared to untreated group.

### 3.5 PLME causes differentially expressed (DE) miRNAs in HeLa cells

**FIGURE 7:**
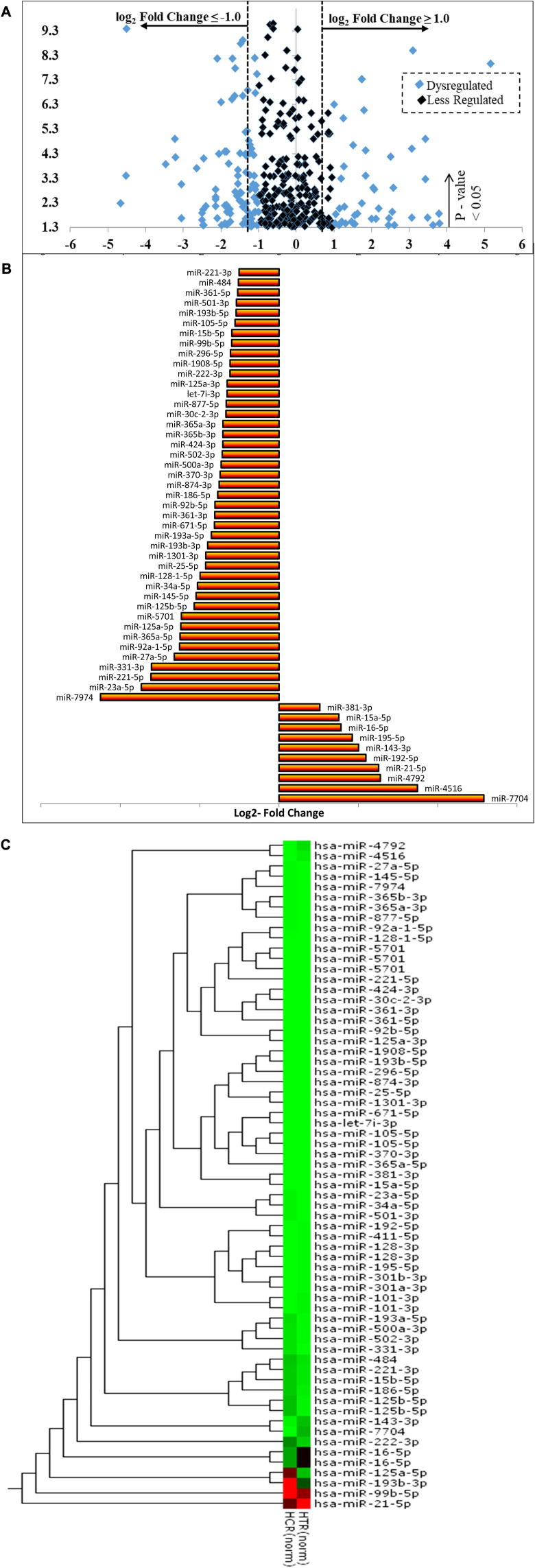
(Continued).

A number of 2588 miRNAs were identified through the cross-analysis of untreated and PLME-treated libraries. *p-*values were generated through the edgeR program and the significant DE of upregulated miRNAs was identified with fold change log2 ≥ 1 whereas downregulated miRNAs were recognized with fold change log2 ≤ −1 and a *p*-value <0.05 was set as a standard to identify the significantly differentially expressed (DE) miRNAs between the groups (Wang et al., 2014). A volcano plot was generated to visualize these miRNA expression levels in association with the *p*-value (Figure 7A). Here, the *x*-axis represented the log_2_ FC (fold change) among these two groups (on a log_2_ scale where the indication of up and downregulation will appear symmetrically). In contrast, the *y*-axis represented the *p*-value on a negative (−log_10_) scale; hence smaller *p*-values will appear higher on the axis. In brief, the first axis (x) indicates the biological impact change, while the second axis (y) indicates statistical evidence or reliability of the change. MiRNAs with statistically significant differential expression were found above the horizontal threshold line of 1.3 (−log_10_ of *p*-value = 0.05). A 458 DE miRNAs were identified from the volcano plot where 47 miRNAs were upregulated, and 87 were downregulated with a *p*-value <0.05. The black dots denote miRNAs between the log2- FC of −1 and 1, while the blue indicates the DE ones.

The further classifies the dysregulated miRNAs based on normalized reads in either condition (untreated or PLME treated) (Figure 7B). Based on the statistical significance (*p* < 0.05), log_2_ FC and normalized reads, the PLME treatment significantly increased the expression of 10 miRNAs (miR-7704, miR-4516, miR-4792, miR-21-5p, miR-192-5p, miR-143-3p, miR-195-5p, miR-16-5p, miR-15a-5p, and miR-381-3p). Out of 10, the highest change was observed for miR-7704 (log_2_ FC = 5.16, *p* < 0.001). However, PLME treatment attenuated the expression of 43 miRNAs (miR-7974, miR-23a-5p, miR-221-5p, miR-331-3p, miR-27a-5p, miR-92a-1-5p, miR-365a-5p, miR-125a-5p, miR-5701, miR-125b-5p, miR-145-5p, miR-34a-5p, miR-128-1-5p, miR-25-5p, miR-1301-3p, miR-193b-3p, miR-193a-5p, miR-671-5p, miR-361-3p, miR-92b-5p, miR-186-5p, miR-874-3p, miR-370-3p, miR-500a-3p, miR-502-3p, miR-424-3p, miR-365b-3p, miR-365a-3p, miR-30c-2-3p, miR-877-5p, let-7i-3p, miR-125a-3p, miR-222-3p, miR-1908-5p, miR-296-5p, miR-99b-5p, miR-15b-5p, miR-15b-5p, miR-105-5p, miR-193b-5p, miR-501-3p, miR-361-5p, miR-484, and miR-221. Out of 43 PLME downregulated miRNAs, the lowest change was observed for miR-7974 (log_2_ FC = −4.50, *p* < 0.001).

The heat map revealed distinct expression patterns in some miRNAs when comparing PLME-treated with untreated control samples (Figure 7C). Referring to the scale bar, the red indicates upregulated while the green indicates downregulated. A dendrogram positioned next to the *y*-axis represents the homological relationship between these miRNAs and this combination is known as hierarchical clustering.

### 3.6 Selection of target gene for GO analysis using PLME-induced miRNAs in HeLa cells

The miRNAs that play the major role were selected based on the criteria of generating a large number of genes with the intersection of at least 3 computational methods consisting of miRanda, Pita, Rnahybrid, TargetScan, and Microtar algorithms with the unison of at least one validated database consisting of TarBase 6.0, MirTarbase 4.5, miRecords, and oncomiRDB. The miRNAs that play a major role were chosen based on the criteria of generating a large number of genes with the intersection of at least three computational methods and unison of at least one validated database (Tables 1, 2). Hence, 5 miRNAs from 10 upregulated genes and 14 miRNAs from 43 downregulated genes were selected.

**TABLE 1:** The selected upregulated miRNAs display the genes targeted by a combination of Computational Algorithm and Validated Database. The computational approaches group consists of miRanda, Pita, Rnahybrid, TargetScan, and Microtar algorithms, while the validated database group consists of TarBase 6.0, MirTarbase 4.5, miRecords, and oncomiRDB.

miRNA	Number of genes predicted with 5 computational approaches	Number of genes identified with experimental 4 validated methods
	≥3 algorithms	≥1 database
miR-143-3p	20	13
miR-195-5p	64	25
miR-16-3p	68	479
miR-15a-5p	122	48
miR-381-3p	31	2

**TABLE 2:** The selected downregulated miRNAs display the number of genes targeted by a combination of Computational Algorithm and Validated Database. The computational approaches group consists of miRanda, Pita, Rnahybrid, TargetScan, and Microtar algorithms, while the validated database group consists of TarBase 6.0, MirTarbase 4.5, miRecords, and oncomiRDB.

miRNA	Number of genes predicted with 5 computational approaches	Number of genes identified with experimental 4 validated methods
	≥3 algorithms	≥1 database
miR-331-3p	119	120
miR-125a-5p	69	60
miR-125b-5p	60	100
miR-34a-5p	78	157
miR-193b-3p	33	189
miR-193a-5p	63	2
miR-186-5p	42	197
miR-370-3p	77	3
let-7i-3p	3	0
miR-222-3p	15	69
miR-296-5p	209	9
miR-15b-5p	111	63
miR-361-5p	13	28
miR-484	59	280

### 3.7 Predicting target gene function in PLME-treated HeLa cells

**FIGURE 8:**
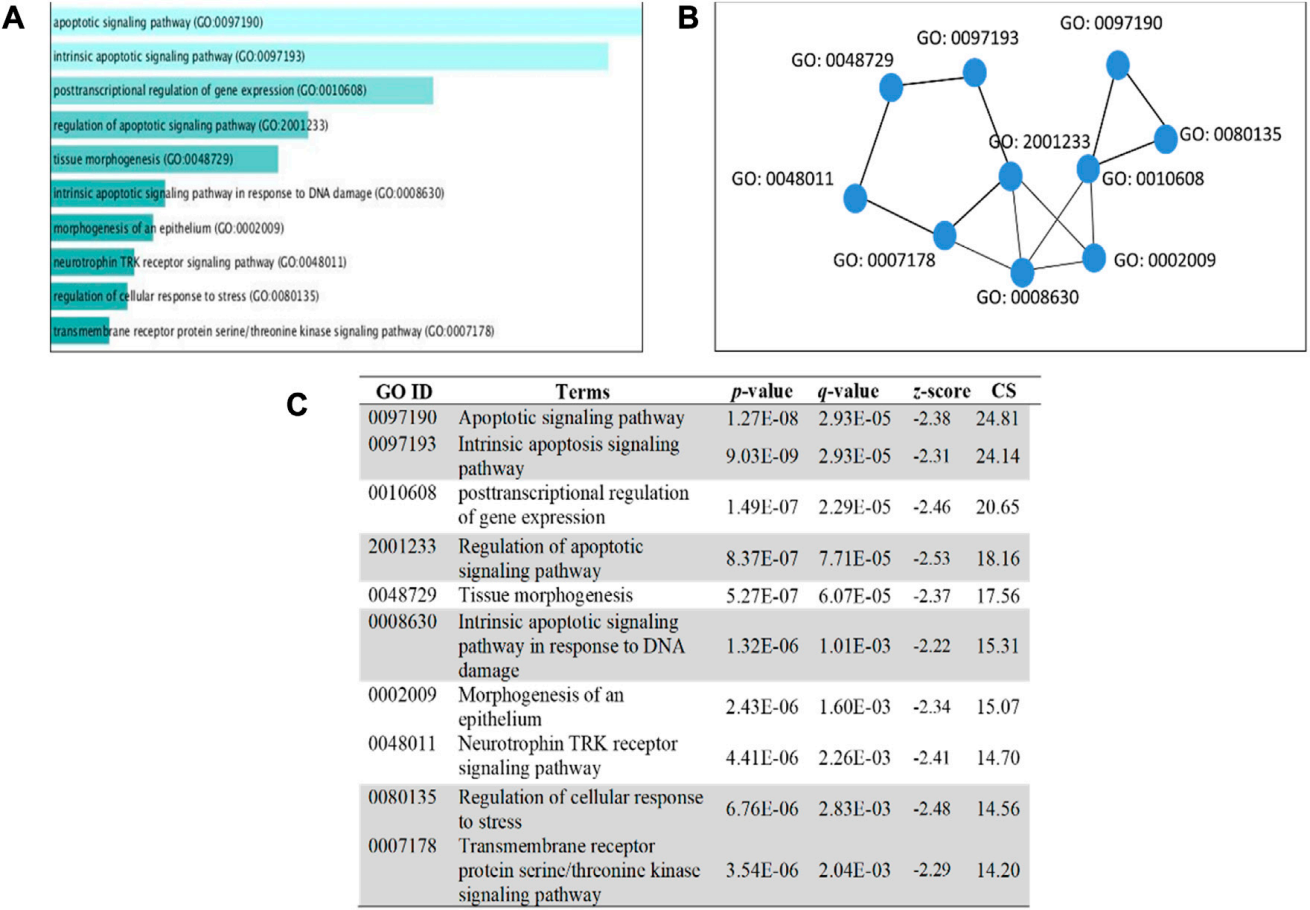
GO term enrichment analyses were performed using Enrichr on DE miRNA genes. **(A)** The top 10 enriched biological processes for DEGs. The horizontal axis represents the number of genes, and the *y*-axis represents the biological process. **(B)** Network view displays the biological process network of the top ten terms based on their gene content similarity. **(C)** The GO term is highlighted to indicate an enrichment score with a *p-*value less than 0.05 concerning apoptosis. The combinational score (CS) was based on *p-*value, *q-*value, and *z-*score.

**FIGURE 9:**
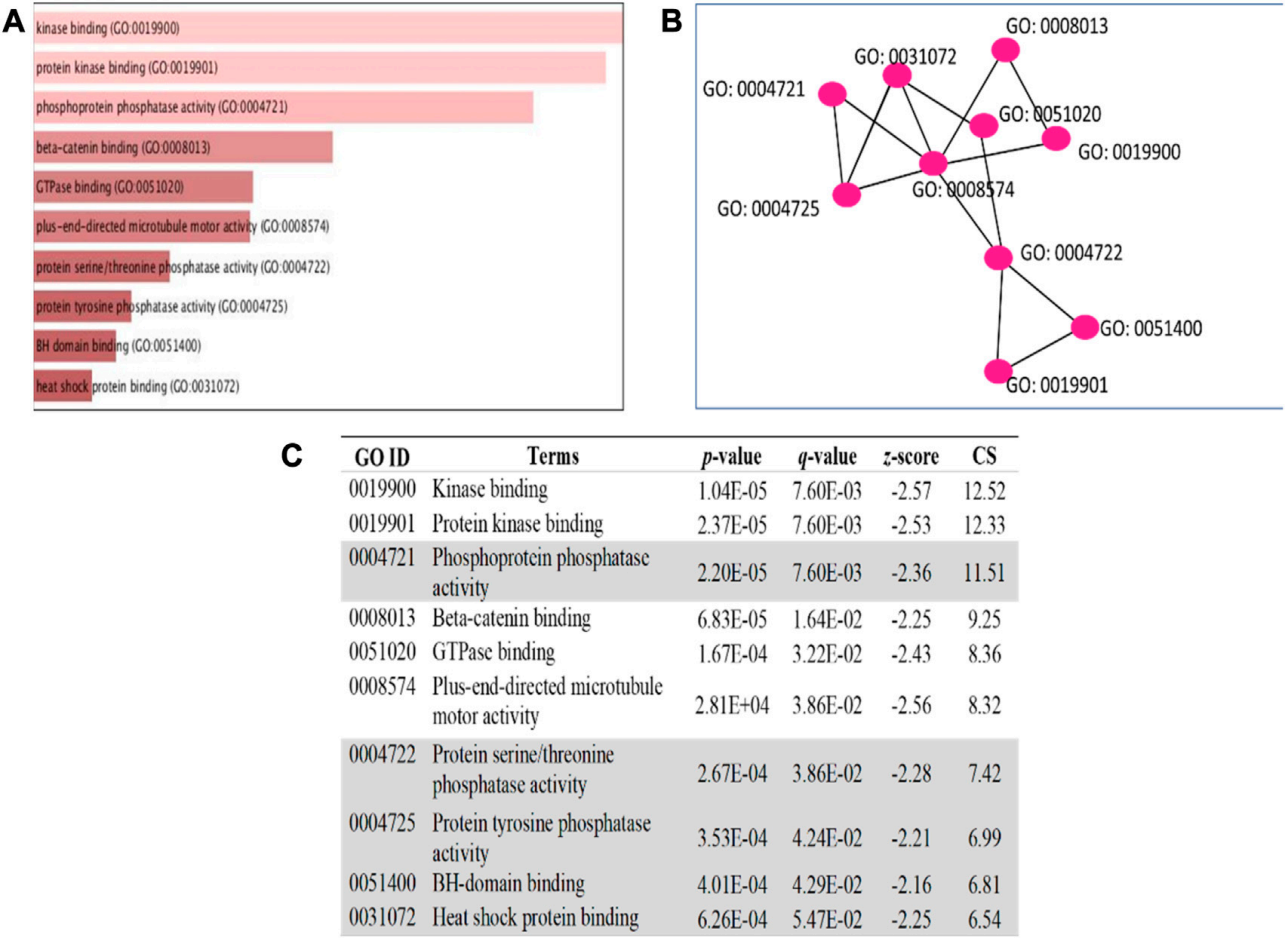
GO term enrichment analyses were performed using Enrichr on DE miRNA genes. **(A)** The top 10 enriched molecular processes for DEGs. The horizontal axis represents the number of genes, and the *y*-axis represents the molecular process. **(B)** Network view displays the molecular process network of the top ten terms based on their gene content similarity. **(C)** The GO term highlighted indicates an enrichment score with a *p*-value less than 0.05 concerning apoptosis. The combinational score (CS) was based on *p*-value, q-value, and z-score.

The 5 selected upregulated miRNAs were found as not enough for meta-analysis by DAVID Bioinformatics; therefore, they have been excluded from network analysis with only 14 downregulated miRNAs to proceed with. A total number of 1826 genes were acquired from the list of 14 miRNAs. Benjamini Hochberg test and FDR (false discovery rate) cutoff (0.05) parameters for multiple correction testing were used. For a large number of gene lists, it is crucial to control the large-scale testing, with preferred FDR. It is used to define the significant test with the expected proportion of false positives ensuring a term to be true positive. DAVID bioinformatics was used to analyze the significant gene ontology terms for biological processes (Table 3) and molecular function (Table 4). The enrichment value (ES) was used to denote the most enriched genes within a single term where a cut-off (1.3) was applied. The terms are then compared in the EnrichR platform to verify those predicted by DAVID corresponding to biological process (Figure 8) and molecular function (Figure 9). Statistical *p-*values, *q-*values, and *z-*values together with their combinational score (SC) were provided as evidence to support the enriched terms. The data is shown as a bar chart, where the extent of the bar signifies the number of gene identifiers in each group. The connections of the GO terms are also denoted with a network-related analysis. The relationship from one GO to another is provided with branches.

**TABLE 3:** The significant gene ontology terms for biological processes adapted from DAVID Bioinformatics related to apoptosis.

GO ID	Term	ES	Count	Benjamini	FDR
GO:0097193	Intrinsic apoptotic signaling pathway	6.02	25	6.40E-07	5.10E-07
GO: 0012501	Programmed cell death	6.02	33	9.30E-03	7.30E-02
GO: 1900740	Positive regulation of protein insertion into mitochondrial membrane involved in apoptosis signalling pathway	6.02	11	1.60E-02	1.60E-01
GO: 0000186	Activation of MAPKK activity	3.53	35	3.20E-01	1.40E-01
GO: 0000165	MAPK cascade	3.53	35	4.70E-01	2.90E-01
GO: 0051403	Stress-activated MAPK cascade	3.02	17	2.50E-02	2.90E-01
GO: 0001836	Release of cytochrome c from mitochondria	2.63	9	1.10E-01	2.40E + 00
GO: 2001244	Positive regulation of intrinsic apoptotic signaling pathway	2.63	10	1.80E-01	4.70E + 00
GO: 0090200	Positive regulation of release of cytochrome c from mitochondria	2.63	9	1.00E+00	7.10E + 00
GO: 0001836	Release of cytochrome c from mitochondria	2.03	9	1.10E-01	2.40E + 00
GO: 0070059	Intrinsic apoptotic signaling pathway in response to endoplasmic reticulum stress	2.03	9	3.60E-01	1.60E + 01
GO: 1903896	Positive regulation of IRE1-mediated unfolded protein response	2.03	3	9.10E-01	9.40E + 01
GO: 0008637	Apoptotic mitochondrial changes	1.81	6	6.80E-01	5.90E+01
GO: 0046902	Regulation of mitochondrial membrane permeability	1.59	7	3.30E-02	4.50E-01
GO: 0001836	Release of cytochrome c from mitochondria	1.59	9	1.10E-01	2.40E + 00
GO: 0051881	Regulation of mitochondrial membrane potential	1.59	5	9.80E-01	1.00E + 02

**TABLE 4:** The significant gene ontology terms for molecular process adapted from DAVID Bioinformatics related to apoptosis.

GO ID	Term	ES	Count	Benjamini	FDR
GO: 0004725	Protein tyrosine phosphatase activity	3.68	21	3.40E-02	4.20E-01
GO: 0008138	Protein tyrosine/serine/threonine phosphatase activity	3.68	10	1.60E-01	4.10E+00
GO:0051434	BH3 domain binding	1.81	4	1.90E-01	5.30E+00

### 3.8 Pathway analysis in PLME-treated HeLa cells

**FIGURE 10:**
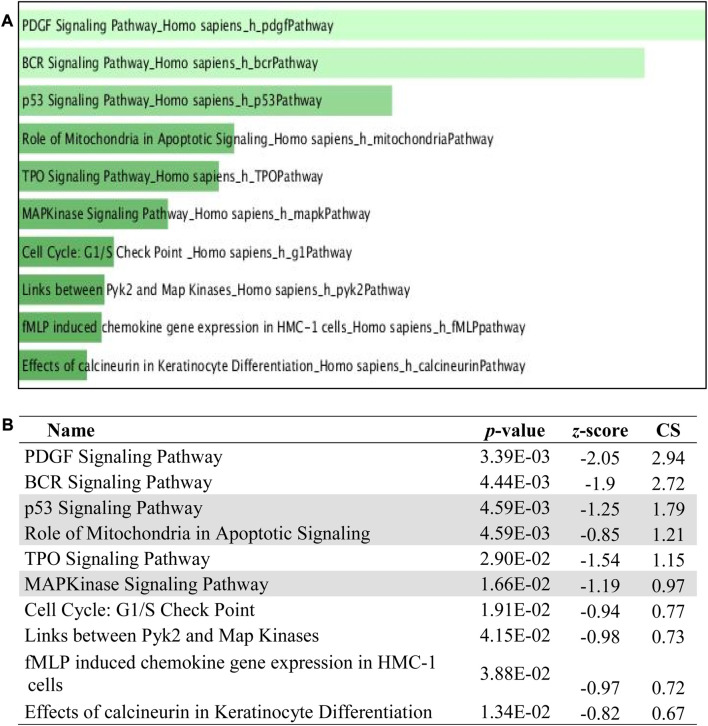
BIOCARTA pathway enrichment analyses were performed using EnrichR on DE miRNA genes. **(A)** The top 10 enriched BIOCARTA pathways. The horizontal axis represents the number of genes, and the *y*-axis represents BIOCARTA pathway names. **(B)** Apoptotic-related pathway terms are highlighted.

**FIGURE 11:**
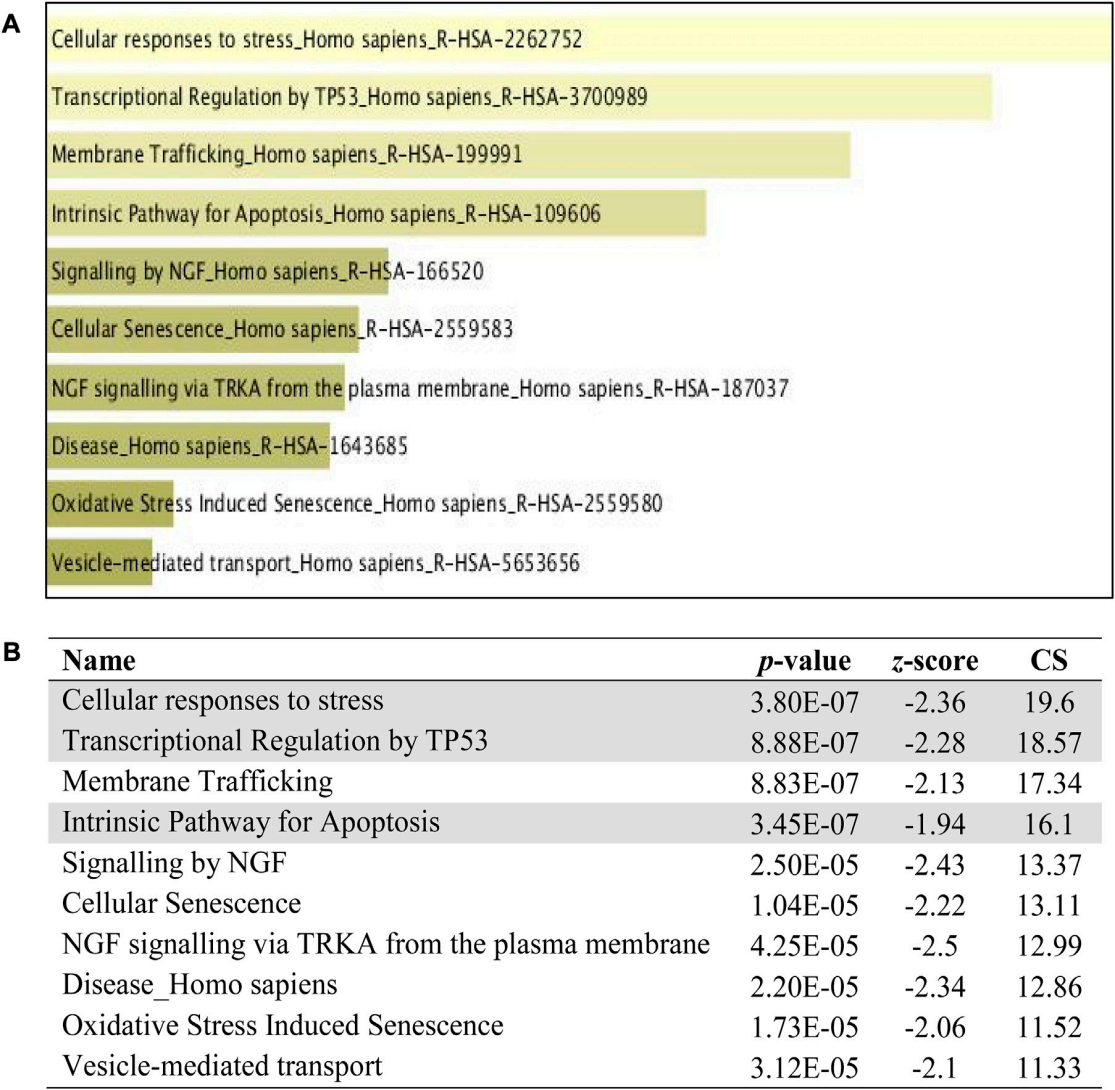
REACTOME pathway enrichment analyses performed using EnrichR on DE miRNA genes. **(A)** The top 10 enriched REACTOME pathways. The horizontal axis represents the number of genes, and the *y*-axis represents the REACTOME pathway names. **(B)** Apoptotic-related pathway terms are highlighted.

The pathway analysis was investigated with three Enrich databases namely,; BIOCARTA and REACTOME. The top 10 ranking data are displayed with the classification pathways in the form of a bar chart with a data label inserted below. All bar charts were obtained from Enrich database. The extent of the bar specifies the total number of gene identifiers computed with a combinational score. Figure 10 reveals the top 10 ranking of BIOCARTA pathways functionally predicted for the downregulated miRNAs. There were mainly three pathways that can be related to apoptosis which are shadowed. Figure 11 displays the top 10 ranking of REACTOME pathways prediction that has three associations with apoptosis. Though, KEGG analysis did not denote any conformity to apoptosis, however, the other pathways did almost familiar prediction to apoptosis.

### 3.9 Phytochemical profile of PLME by LC-ESI-MS/MS spectrometry analysis

**FIGURE 12:**
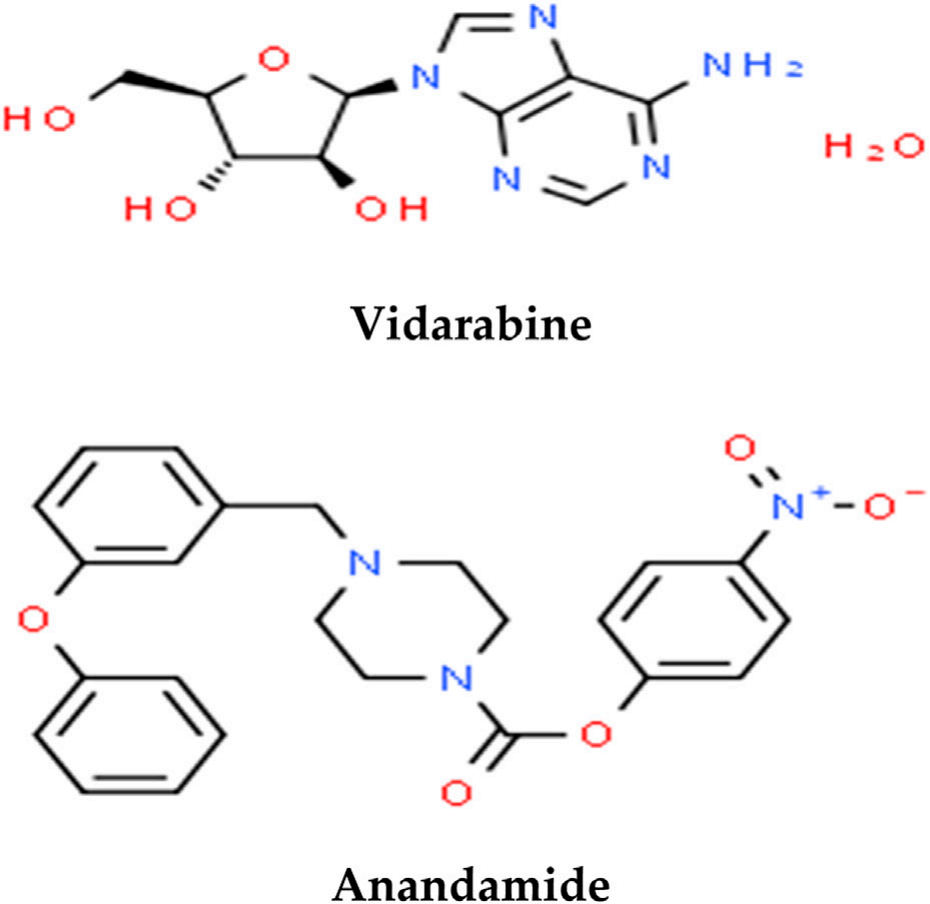
Anticancer phytochemical compounds found in the methanol extract of Polyalthia longifolia Leaf (MEPL) were detected using ultra-high-performance liquid chromatography (UHPLC) equipped with the chemical library.

The LC-ESI-MS/MS spectrometry analysis was performed to explore the phytochemical profile of PLME. The LC-ESI-MS/MS spectrometry analysis of the MEPL showed the presence of anticancer phytochemicals in MEPL. Among these identified compounds in MEPL extract, Vidarabine, and Anandamide. The chemical structures of the anticancer compounds found in MEPL are presented in Figure 12.

## 4 Discussion

The current study was conducted to analyze the differences in miRNA and their apoptotic gene profiles between untreated and PLME-treated HeLa cells. In our previous investigation, PLME demonstrated cytotoxicity against HeLa cells with half-maximal cytotoxicity activity (IC_50_) of 22.00 µg/mL (Vijayarathna et al., 2017b). PLME has also been indicated to be involved in plasma membrane disruption, cell cycle arrest, membrane potential loss in mitochondrial, generation of reactive oxygen species, and DNA fragmentation (Vijayarathna et al., 2017a). Hence, the current study is conducted to identify the role of miRNAs associated with the induction of apoptotic cell death in HeLa cells.

### 4.1 Assessment of total RNA purity and integrity

The advancement of RNA sequencing begins with 1) isolating total RNA with RIN >9, 2) the selection of gel electrophoresis bands conformity to miRNA upon library amplification, and 3) quality assessment of purified cDNA molecules prior to sequencing. The downstream analysis reckons the subject of RNA quality and integrity (Cseke et al., 2011). Total RNA isolated from HeLa cells (untreated and PLME treated) was instantly measured for its yield and purity after purification. A ratio reading which is not within the range of 2.0–2.2 is regarded as poor quality (Glasel, 1995). The RNA concentration is contemplated to *A*_*260*_ reading with the intended conversion factor based on the extinction constant of RNA (*A*_*260*_ of 1.0 = 40 µg/mL). The readings obtained from the samples fall in accordance with Sambrook *et al.* (Sambrook et al., 1989), in which the publication stated that a pure RNA exists when the ratio of (*A*_*260:*_ *A*_*230*_) is measurably equal to (*A*_*260:*_ *A*_*280*_), on the condition that the value is greater than 1.8. However, absorbance ratios of high RNA purity alone do not pay to be indicative of sample quality. For that account, a second assessment method by electrophoresis (Figure 3B) was performed using gel run with RNA stained ethidium bromide (EtBr) (Le Pecq and Paoletti, 1966; Vendrely et al., 1968; Lehrach et al., 1977). Intact RNA run on the gel will exhibit a single column band of 28S and 18S in sharp and clear visual as well as 28S rRNA appearing twice as intense as 18S rRNA band with 5kb and 2kb respectively. A 28S:18S ratio of 2:1 is applicable as a reference point for intact RNA (Beneyto et al., 2009).

A more precise method is referred to Agilent 2100 Bioanalyzer results (Figure 3C). Combining RNA area peaks of 18S and 28S, then dividing the area of 18S RNA peak into the area of 28S RNA peak will result in the rRNA ratio of 28S:18S. The RIN is addressed with a scale from 0 to 10, with 10 being the maximum RNA integrity whereas the RNA concentration is determined by measuring the area under the entire RNA electropherogram. The ladder bearing the concentration/area ratio is utilized to convert the area values into concentration values (Lightfoot, 2002). The HeLa cell RNA samples procured from this study (Figure 3C), are observed to be of relatively good purity with high RIN.

### 4.2 cDNA library preparation for high-throughput small RNA sequencing

Benefits from next-generation sequencing include achieving fast characterization and quantification of transcriptomes in RNA-seq. The steps in RNA-seq progress with direct sequencing of complementary DNA (cDNA). The converted cDNA libraries are then amplified and run on gel where desired band sizes of 135 bp to 160 bp were excised and purified for quality and concentration assessment. The sizes of base pairs are specified by using electrophoregrams from Agilent Bioanalyzer. Single peaks are noticed as protrusion of the *x*-axis at 141 bp and 137 bp (Figure 4A) denoting the occurrence of miRNAs. No other peaks were observed except for the markers detected at 15 bp and 1,500 bp, hence the libraries are affirmed suitable for sequencing analysis.

The concentration of the libraries has to be cardinally enumerated to achieve optimum cluster densities whenever using Illumina sequencing platforms. The outcome is displayed in a standard curve (plot of C_t_ values/crossing points of diverse standard dilutions against log of amount of standard) where it is created with standard series of six dilution concentrations (Figure 4B). The concentrations of unknown targets are quantified (Figure 4D) by referring to the C_t_ values (Figure 4C) of the unknowns to the equation obtained from the standard curve plot. In parallel, the concentrations of HeLa cells from two different conditions are measured for validity before sequencing.

### 4.3 Analysis of small RNA-Seq

Two of the key parameters necessary for examining raw sequencing precision are base quality and percentage of GC content (Guo et al., 2014). A typical strategy employed for the percentage of GC content is also noted as an indicator of good reads. A high GC (>60%) content will point to the presence of rRNA in the sample or contamination by bacteria or fungi (organisms with higher GC). A lower GC on the contrary will indicate the presence of mRNA poly A-tails (Delhomme et al., 2014). Filtered data is submitted for a QC assessment by FastQC and the non-strand specific RNA-seq reads should maintain an on-average-equal amount of GC to AT within any position of reads. Figure 5B clearly exhibits the content of GC and AT to be within a closer range that justifies the quality of data for studying differential expression. On the other hand, a graphical account of base quality is to sketch the base Q score against the cycle plot. The Q score has been a defining accuracy key for the utility of high-throughput Sanger sequencing (Ewing and Green, 1998). The Q score quality values facilitate a fundamental trait for filtering low-confidence sequences from the sequence reads (Holt and Jones, 2008; Bokulich et al., 2013).

Figure 5C displays the score distribution statistic of Q20 and Q30. Based on the raw sequence collected, it can confidently state more than 90% of the base calls possess an accuracy of 99.0%–99.9% overall. A threshold of Q20 is widely accepted as it corresponds to a base call error of 1 in 100, which is approximately the inherent technical error rate of the Illumina sequencing platform.

The next step on Illumina sequencers requires the linkage of the quality base pair to its position of read called mapping. As soon as the quality reads are evaluated and regarded as adequate or have been filtered to agreeable requirements, the reads are mapped using Bowtie 1 for its alignment search. Bowtie aligns reads back to its reference transcriptome. After stringent filtering, the remaining reads are used as a query to map against genomic RNA data that encompasses rRNA, tRNA, mRNA, and miRNA (Zhang et al., 2015). The sequential mapping of RNA is illustrated in Figure 6. Only the reads mapping to mirBase v.21 is mapped against GrCH38, the human genome while those that failed to map are classified as others.

### 4.4 Quantification of transcript using miRDeep2

MiRNAs of known sequences are selected in reference to miRBase v.21 and the unannotated reads were scrutinised using miRDeep2 to separate the known miRNAs from the novel ones (Zhang et al., 2015). miRDeep2 is integrated computation software utilized in annotating miRNAs from raw RNA-seq reads as well as quantifying their expressions. The output of miRDeep2 is classified with three embedded modules, namely, mapper, miRDeep2, and quantifier. The expression of the raw read counts should be subjected to normalization since these raw reads alone are not adequate for comparison of expression levels among samples. The read counts are susceptible to various interferences such as transcript length, total number of reads, and sequencing biases (Conesa et al., 2016). On that account, to receive an equal comparison expression from untreated and treated HeLa cells, TPM (transcript per million) was utilized.

### 4.5 Quantification of differentially expressed miRNAs

The edgeR program represents a negative binomial model to estimate biological and technical replicates where they were used in parallel to miRDeep2 analyses (Plieskatt et al., 2014). Approximately 2588 numbers of miRNAs are listed in the miRDeep2 analysis with the read counts in TPMs. MiRNAs with the extreme fold change in their expression ([log_2_ (fold change)] ≥ 1.0) and ([log_2_ (fold change)] ≤ −1.0) were graphed into a volcano plot and graph bar (Figures 7A, B) for the identification of DE miRNAs based upon their fold change and *p*-values. A heat map was generated to display DE miRNAs between samples while the embedded hierarchical clustering diagram explains the relationship between those miRNAs (Figure 7C). Conclusively, the selection of dysregulated miRNAs was recognized to be 53 miRNAs based on their extreme fold change where; 10 upregulated and 43 downregulated are investigated for their gene targets and functional studies.

### 4.6 MiRNA target prediction

The expression of a miRNA is decisively defined by the genes that it targets and also by the manifestation of the operated genes. Annotating these DE miRNAs stipulates direct target genes recognition and interaction (Papadopoulos et al., 2009). The progression to determine the relationship between miRNAs and their gene targets is still deliberately tedious and slow caused by factors such as low expression of miRNAs, lower stability, and tissue specificity. Hence, numerous computation prediction applications have been seen to grow actively to help predict miRNA gene targets utilizing statistical algorithms based upon the criteria of miRNA to the 3’ UTRs of transcripts (Lee et al., 1993). The miRGate database merges novel prediction miRNA-mRNA pair altogether with prominently valid algorithmic programs considering miRanda, Pita (Kertesz et al., 2007), RNAhybrid (Krüger and Rehmsmeier, 2006) or MicroTar (Thadani and Tammi, 2006) among others. Moreover, experimentally validated miRNA-mRNA pairs are also accounted as features of high reliability with regard to gene tool prediction. The miRNAs (from up and downregulated) were further selected based upon the highest gene counts obtained from the intersections of more than three prediction methods along with at least one experimentally validated program (Tables 1, 2). When more programs were found to predict these genes, the chances of that miRNA targeting these genes seems reliable to investigate their underlying functions.

### 4.7 Gene ontology and pathway analysis

It can be deduced that the PLME treated HeLa cells have 14 downregulated miRNAs. Therefore, the inference deriving from this state of miRNAs is that the downregulated miRNAs will no longer target their specific genes, thereby increasing the density of genes to perform their functions without inhibition. The enrichment analysis is elucidated by a technique termed called gene ontology (GO) (Ashburner et al., 2000). Utilizing a single database to predict target enrichment would mislead the concept of prediction, not to mention the very fact that each database applies different statistical models. The DAVID statistical methods emphasize modified Fisher’s exact test (EASE score) to investigate the significance of gene-term enrichment with the use of multiple testing correction techniques (Bonferroni, Benjamini, and FDR) to globally correct the enrichment family-wide *p*-values (Huang et al., 2007). Inversely, EnrichR computes three types of enrichment scores to assess; the Fisher’s exact test (a test that is implemented in most gene enrichment programs), *z*-score (based on nonconformity from the predictable rank by Fisher exact statistical test), and pooled score (multiplies log *p*-value computed with Fisher exact test by the *z*-score) (Chen et al., 2013). DAVID analysis of the genes involved in biological processes (Table 3) indicated chiefly overrepresented GO terms related to intrinsic apoptosis signaling pathway, activation of MAPK cascade, the release of cytochrome *c* from mitochondria, regulation of ∆Ψ_m_ and intrinsic apoptosis in response to the endoplasmic reticulum (ER). Corroboratively, the EnrichR GO term of biological process (Figure 8) validates DAVID performance by annotating intrinsic apoptosis signaling pathway in response to DNA damage, apoptosis pathway signaling, and regulation of cellular response to stress. The most enriched GO terms in molecular function (Table 4) annotated by DAVID are protein tyrosine phosphatize activity, protein tyrosine/serine/threonine phosphatase activity, and BH-3 domain binding. Similar terms were observed for Enrichr (Figure 9) following gene enrichment where an extra addition in terms for protein kinase and heat shock protein binding. The enriched genes decidedly suggest their function in phosphatase activities. Consideration should be given as not all phosphate addition and detachments correspond to enzyme activation or inhibition in a functional regulation. Moreover, phosphorylation is vital for intracellular proteins such as to carry out pro-apoptotic and anti-apoptotic especially surrounding the caspases and BCL-2 family proteins cascade (Cai et al., 1998; Choi et al., 2002).

The term pathway analysis is widely utilized in a prominent manner during the course of publications (Green and Karp, 2006) inclusively with GO terms. The typical manner, by which cells interact with the surrounding cues and signal among themselves, is crucial to comprehend and interpret the function and controlled regulation of pathways. The pathway analysis from BIOCARTA and REACTOME (Figures 10, 11) revealed an apoptosis signaling pathway with a relation to mitochondria, p53, cellular stress response, MAPKinase signaling, and the intrinsic apoptosis pathway which coincides with the GO term identified earlier. An inference was made on the premises of the GO and the pathway analysis that PLME enriched these genes in HeLa cells by downregulating 14 miRNAs with a possible notion to apoptosis pathways via caspase, cytochrome *c*, mitochondria permeability, MAPK cascade activity, ER-stress, and p53 activation.

### 4.8 Chemical profiles of PLME

The screening using UHPLC analysis equipped with the chemical library was done to examine the chemical profiles of the eluted bands of PLME chromatogram in an attempt to identify the bioactive chemical compounds, that could be responsible for the observed cytotoxicity against HeLa cells. As seen in Figure 12, this led to the identification of the two compounds. Interestingly, the presence of Vidarabine (Arif et al., 2004; Sneader, 2005) and Anandamide (Adinolfi et al., 2013) compounds in *P. longifolia* leaf extract, which was previously reported to inducing cancer cells cytotoxicity, might have contributed to the observed cytotoxicity properties of the tested extract in this study. Structurally, both Vidarabine and Anandamide compounds have phenolic rings, which might be involved in the pro-oxidant features mainly responsible for apoptosis-based cell cytotoxicity (Nandi et al., 2007). It is commonly reported that the cell cytotoxicity related to some phenolic compounds is facilitated by their oxidative and pro-oxidant activities, which can quicken the oxidative damage *in vitro*, either to DNA or proteins. However, in-depth isolation and identification study is compulsory on these bioactive compounds of *P. longifolia* leaf extract.

### 4.9 The postulated mechanism of PLME inducing apoptosis in HeLa cells

Therefore, the effects of PLME treatment on HeLa cells specify that the therapy enhanced the cytotoxicity activity of PLME towards HeLa cells in terms of apoptotic cell death by regulating key miRNAs. These Proposed models of PLME extract mechanism of action for the HeLa cell cytotoxicity are consistent with the previous *in vitro* findings of this extract against HeLa cells as reported by Vijayarathna et al. (2017b) and Cilwyn-Shalitha and Sasidharan (2023). Vijayarathna et al. (2017b) reported that the PLME treatment against HeLa cells resulted in mitochondrial potential depolarization, cell cycle arrest, loss of ΔΨm, generation of ROS, and DNA fragmentation, which might be related to the pro-oxidant activity of PLME to increase the oxidative stress leads to the induction of DNA damage and the loss of DNA repair capacity. Besides, they also reported that the PLME treatment also increased the level of pro-apoptotic BAX, BAD, caspase-3, p53, and p21 significantly while causing a decrease in the expression of anti-apoptotic proteins, BCL-2 and BCL-w. In addition, Cilwyn-Shalitha and Sasidharan (2023) recently reported that the effects of PLME treatment on HeLa tumor cells that reduced the Ki-67, VEGF, and CD31 proteins expressions might facilitate inhibition of the growing HeLa tumor cells by apoptosis induction. In conclusion, the PLME-induced p53 mediated apoptosis, cell cycle arrest, and mitochondrial potential depolarization by modulating the redox status and regulating key miRNAs in the HeLa cells.

## 5 Conclusion

In summary, the results of the present study indicate that the treatment of HeLa cells with PLME enhanced the cytotoxicity activity of PLME towards HeLa cells in terms of cell proliferation, survivability, and apoptotics by the downregulation of key miRNAs. Potential target prediction computational methods linked apoptosis mechanisms to these downregulated miRNAs. The development of green natural remedies may assist deeper understanding of the apoptotic response toward cancer serving as potential anticancer therapy through the identification of miRNAs. Further study should be conducted to validate the function of the highly regulated miRNA in HeLa by PLME. Besides, the validated potential miRNA also can be tested in an *in vivo* tumor preclinical animal model as a targeted miRNA source for gene therapy.

## Data Availability

The original contributions presented in the study are included in the article/Supplementary Material, further inquiries can be directed to the corresponding author.
